# Bi-allelic *GAD1* variants cause a neonatal onset syndromic developmental and epileptic encephalopathy

**DOI:** 10.1093/brain/awaa085

**Published:** 2020-04-13

**Authors:** Nicolas Chatron, Felicitas Becker, Heba Morsy, Miriam Schmidts, Katia Hardies, Beyhan Tuysuz, Sandra Roselli, Maryam Najafi, Dilek Uludag Alkaya, Farah Ashrafzadeh, Amira Nabil, Tarek Omar, Reza Maroofian, Ehsan Ghayoor Karimiani, Haytham Hussien, Fernando Kok, Luiza Ramos, Nilay Gunes, Kaya Bilguvar, Audrey Labalme, Eudeline Alix, Damien Sanlaville, Julitta de Bellescize, Anne-Lise Poulat, EuroEpinomics-RES consortium AR working group, Ingo Helbig, Sarah von Spiczak, Stephanie Baulac, Nina Barisic, Rudi Balling, Hande Caglayan, Dana Craiu, Renzo Guerrini, Karl Martin Klein, Carla Marini, Hiltrud Muhle, Felix Rosenow, Jose M Serratosa, Katalin Sterbova, Yvonne Weber, Ali-Reza Moslemi, Holger Lerche, Patrick May, Gaetan Lesca, Sarah Weckhuysen, Homa Tajsharghi

**Affiliations:** a1 Genetics Department, Lyon University Hospital, Lyon, France; a2 Institut NeuroMyoGène CNRS UMR 5310 - INSERM U1217 Université de Lyon, Université Claude Bernard Lyon 1, Lyon, France; a3 Department of Neurology, University of Ulm, Ulm, Germany; a4 University of Tübingen, Department of Neurology and Epileptology, Hertie Institute for Clinical Brain Research, Tübingen, Germany; a5 Human Genetics Department, Medical Research Institute, Alexandria University, Alexandria, Egypt; a6 Genome Research Division, Human Genetics Department, Radboud University Medical Center Nijmegen, Nijmegen, The Netherlands; a7 Radboud Institute for Molecular Life Sciences, Geert Grooteplein Zuid 10, 6525KL Nijmegen, The Netherlands; a8 Center for Pediatrics and Adolescent Medicine, University Hospital Freiburg, Freiburg University Faculty of Medicine, Freiburg, Germany; a9 Neurogenetics Group, VIB-Center for Molecular Neurology, University of Antwerp, Antwerp, Belgium; a10 Department of Pediatric Genetics, Istanbul University-Cerrahpasa, Medical Faculty, Istanbul, Turkey; a11 Department of Pathology, University of Gothenburg, Sahlgrenska University Hospital, Sweden; a12 Department of Paediatric Neurology, Ghaem Medical Centre, School of Medicine, Mashhad University of Medical Sciences, Mashhad, Iran; a13 Pediatrics Department, Faculty of Medicine, Alexandria University, Alexandria, Egypt; a14 Genetics Research Centre, Molecular and Clinical Sciences Institute, St George’s, University of London, Cranmer Terrace, London SW17 0RE, UK; a15 Innovative medical research center, Mashhad branch, Islamic Azad University, Mashhad, Iran; a16 Universidade de Sao Paulo Faculdade de Medicina, Sao Paulo, SP, Brazil; a17 Department of Genetics, Yale Center for Genome Analysis (YCGA), Yale University, School of Medicine, New Haven, Connecticut; a18 Department of Pediatric Clinical Epileptology, Sleep Disorders and Functional Neurology, ERN EpiCARE, University Hospitals of Lyon, Lyon, France; a19 Department of Pediatric Neurology, Lyon University Hospital, Lyon, France; a20 Luxemburg Centre for Systems Biomedicine, University of Luxembourg, Belvaux, Luxembourg; a21 Department of Neurology, University Hospital Antwerp, Antwerp, Belgium; a22 School of Health Sciences, Division Biomedicine, University of Skovde, Skovde, Sweden

**Keywords:** GAD1, suppression-burst, hypsarrhythmia, arthrogryposis, omphalocele, cleft palate

## Abstract

Developmental and epileptic encephalopathies are a heterogeneous group of early-onset epilepsy syndromes dramatically impairing neurodevelopment. Modern genomic technologies have revealed a number of monogenic origins and opened the door to therapeutic hopes. Here we describe a new syndromic developmental and epileptic encephalopathy caused by bi-allelic loss-of-function variants in *GAD1*, as presented by 11 patients from six independent consanguineous families. Seizure onset occurred in the first 2 months of life in all patients. All 10 patients, from whom early disease history was available, presented with seizure onset in the first month of life, mainly consisting of epileptic spasms or myoclonic seizures. Early EEG showed suppression-burst or pattern of burst attenuation or hypsarrhythmia if only recorded in the post-neonatal period. Eight patients had joint contractures and/or pes equinovarus. Seven patients presented a cleft palate and two also had an omphalocele, reproducing the phenotype of the knockout *Gad1*^−/−^ mouse model. Four patients died before 4 years of age. *GAD1* encodes the glutamate decarboxylase enzyme GAD67, a critical actor of the γ-aminobutyric acid (GABA) metabolism as it catalyses the decarboxylation of glutamic acid to form GABA. Our findings evoke a novel syndrome related to GAD67 deficiency, characterized by the unique association of developmental and epileptic encephalopathies, cleft palate, joint contractures and/or omphalocele.

## Teaser

Chatron *et al.* describe a novel syndrome caused by bi-allelic loss-of-function mutations in *GAD1*, the gene encoding the GABA synthetic enzyme GAD67. The syndrome is characterized by the unique association of developmental and epileptic encephalopathy, cleft palate, joint contractures and/or omphalocele.

## Introduction

Proper function of the brain is governed by the dense network of neuronal connections. Inhibitory interneurons play a crucial role in controlling network activity. Glutamate decarboxylase (GAD) catalyses the conversion of the excitatory neurotransmitter l-glutamic acid to γ-aminobutyric acid (GABA), the main inhibitory neurotransmitter in the mammalian CNS. In vertebrates, GAD exists in two isoforms, GAD67 and GAD65, encoded by two independent genes *GAD1* and *GAD2*, respectively. While GAD67 (MIM#605363) is ubiquitously expressed and uniformly distributed throughout the neuron and preferentially synthesizes cytoplasmic GABA (Dirkx *et al.*, 1995; Laprade and Soghomonian, 1995), GAD65 (MIM#138275) is localized in nerve terminals and regulates the synthesis of GABA for vesicular release (Soghomonian and Martin, 1998). GAD67, the major isoform at embryonic stages (Asada *et al.*, 1997; Soghomonian and Martin, 1998), has an essential role in neuronal development and synaptogenesis, as well as in normal development of the palate and foetal movements (Asada *et al.*, 1997; Oh *et al.*, 2010).

The importance of GAD67 for neuronal and non-neuronal development has been shown in different animal models. *Gad1*^−/−^ mice die at birth with cleft palate and hypoxia and exhibit reduced levels of GABA in the cerebral cortex (Asada *et al.*, 1997; Condie *et al.*, 1997). About half of *Gad1*^−/−^ mice also show omphalocele (Saito *et al.*, 2010). Their early mortality, however, precludes the evaluation of any neurological phenotype. Homozygous knockdown of *gad1* in zebrafish also results in craniofacial defects and early lethality (Zhang *et al.*, 2017). However, a recent study managed to bypass the early developmental stages through the use of a photo-activatable morpholino oligonucleotide, allowing zebrafish to develop normally. The animal showed increased electrophysiological brain activity and behavioural correlates of seizures in mutants (O’Connor *et al.*, 2019).

In humans, *GAD1* has been described as a susceptibility gene for schizophrenia (Addington *et al.*, 2005; Straub *et al.*, 2007; Zhao *et al.*, 2007). The role of *GAD1* with monogenic disorders is, however, less well established. *GAD1* has been suggested as a candidate gene for autosomal recessive spastic cerebral palsy (McHale *et al.*, 1999), a permanent non-progressive central motor dysfunction that affects muscle tone, movement and posture, as a result from defects in the developing CNS (Hughes and Newton, 1992; Rosenbaum *et al.*, 2007; Rosenbloom, 2007). Subsequently, a homozygous missense variant in *GAD1* leading to a p.(Ser12Cys) substitution in GAD67, has been identified in a single family with four affected siblings with autosomal recessive cerebral palsy spastic quadriplegic type 1 (CPSQ1) (MIM#603513) (Lynex *et al.*, 2004). The clinical characteristic included a spastic diplegia with mild hypertonia in the arms, and moderate-to-severe intellectual disability. Two affected siblings had flexion contractures at the knees. The report did not mention any seizures, dysmorphic or non-neurological features. However, no functional studies were performed to validate this finding. In addition, non-syndromic cleft lip with or without cleft palate (NSCLP), one of the most common craniofacial malformations, has been associated with GAD67 in the Japanese population (Kanno *et al.*, 2004).

Here, we report 11 affected individuals from six unrelated families, with a severe neurodevelopmental phenotype consisting of early-onset epilepsy, intellectual disability, cleft palate and a variable association of pes equinovarus, arthrogryposis multiplex congenita, scoliosis and omphalocele due to bi-allelic loss-of-function variants in *GAD1*.

## Materials and methods

### Patients and genetic analysis

**Figure 1:**
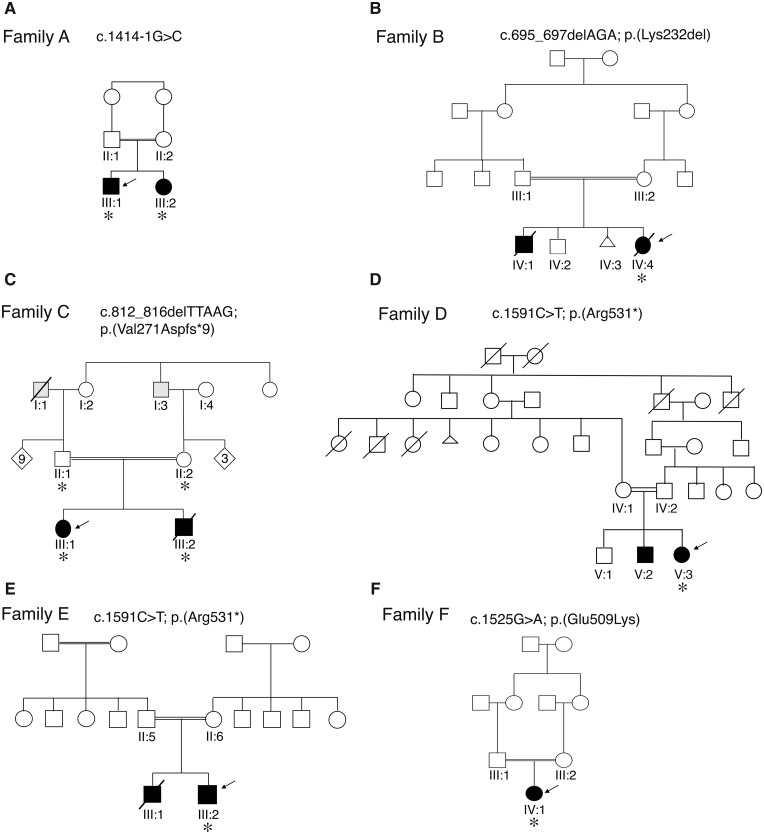
**Pedigrees of the families with *GAD1* variants**. Pedigrees of Families A–F. In the pedigree, squares = males; circles = females; open symbols = unaffected family members; slash = deceased. Index cases (arrows) and family members who were analysed by next generation sequencing (asterisks) are indicated. WES was performed for Families A, B, E and F and WGS for Families C and D. The grey symbols in the pedigree C indicate adult-onset epilepsy without intellectual disability in two family members. Affected individuals are represented with black shaded symbols. The *GAD1* variants identified in each family are indicated.

Eleven patients from six unrelated families of Algerian (Family A), Egyptian (Family B), Turkish (Families C and E), Iranian (Family D) and Brazilian (Family F) origin were included in this study (Fig. 1). The respective families were identified through GeneMatcher (Sobreira *et al.*, 2015). Written informed consent for genetic testing and photographic material were obtained from the parents or legal guardians. The study was conducted in accordance with the Declaration of Helsinki and it was approved by the relevant institutional review boards in Netherlands, Iran, Egypt, Germany, Sweden, Turkey and Brazil.

Molecular genetic analyses were performed in different research and diagnostic centres. Blood samples were obtained from the patients, their parents and unaffected siblings in some families. Whole-exome sequencing (WES) or whole-genome sequencing (WGS) was performed on Patients A-III:1 and A-III: 2 from Family A, Patient B-IV:4 from Family B, Patients C-III:1 and C-III:2 and their parents (Subjects C-II:1 and C-II:2) from Family C, Patient D-V:3 from Family D, Patient E-III:2 from Family E and Patient F-IV:1 from Family F, independently. Detailed genetic methods are provided in the Supplementary material.

*GAD1* variants identified in the patients and their family members were validated by bidirectional Sanger sequencing, for each family, independently. PCR primers are available on request. *GAD1* transcript NM_000817.2 was used for variant nomenclature.

### Complementary DNA analysis in Family A

To study the effect on splicing of the c.1414-1G>C *GAD1* variant identified in Family A reverse transcription of RNA extracted with the RNeasy^®^ Mini Kit (Qiagen) from Epstein-Barr virus–immortalized lymphoblastoid cells of both parents (Subjects A-II:1 and A-II:2) was performed using the Expand™ RT kit (Roche Applied Science), according to the manufacturer’s protocol. A nested PCR approach was used to amplify exons 14 to 18 (primer sequences are available in the Supplementary material). TOPO TA Cloning pCR™2.1-TOPO^®^ vector kit (Thermo Fischer Scientific) was used to separate the different PCR products that were finally sequenced with a 3500 genetic analyzer (Applied Biosystems).

### Generation of p.Lys232del-GAD67 in cell-free protein expression assay in Family B

Cell-free protein expression assay was performed to study the impact of the c.695_697delAGA, p.(Lys232del) variant identified in Family B on expression levels of GAD67. The human full-length wild-type *GAD1* cDNA fragment (NM_000817.2) was cloned into a pRSET-A express cloning vector followed by introduction of the c.695_697delAGA variant by QuikChange II (Agilent Technologies). Wild-type and mutant-*GAD1* constructs were sequenced to verify the complete GAD67 coding sequence and correct introduction of the desired mutation. Recombinant wild-type and p.Lys232del-GAD67 were expressed from the constructs using EasyXpress™ Insect Cell Protein Synthesis Kit (Invitrogen), according to the manufacturer’s protocol.

### Immunoblot analysis of wild-type and p.Lys232del-GAD67

Immunoblotting was performed using the N-terminally acetylated recombinant wild-type and p.Lys232del-GAD67 proteins. Lysates were prepared as previously described (Olive *et al.*, 2015), run on 12% NuPAGE^®^ Bis-Tris gels and transferred onto polyvinylidene difluoride (PVDF) membranes, using a chromogenic western blot immunodetection kit, WesternBreeze™ (Invitrogen), according to the manufacturer’s instructions. The blot was probed with the primary antibodies (anti-GAD67, 1:1000) (Sigma-Aldrich) overnight at 4°C.

### Missense variant 3D modelling

GAD67 protein modelling and prediction of the effects of the p.(Glu509Lys) variant were generated using bioinformatic tools from HOPE (Venselaar *et al.*, 2010). The model was generated based on a homologous structure of yeast GAD67 (PDB ID: 2OKJ) and built using the Yasara/WHAT IF Twinset. Structural information was collected using information from WHAT IF Web services, the UniProt database and the Reprof software.

### Data availability

The authors confirm that the data supporting the findings of this study are available within the article and its Supplementary material.

## Results

### Clinical characteristics of affected individuals

**Figure 2:**
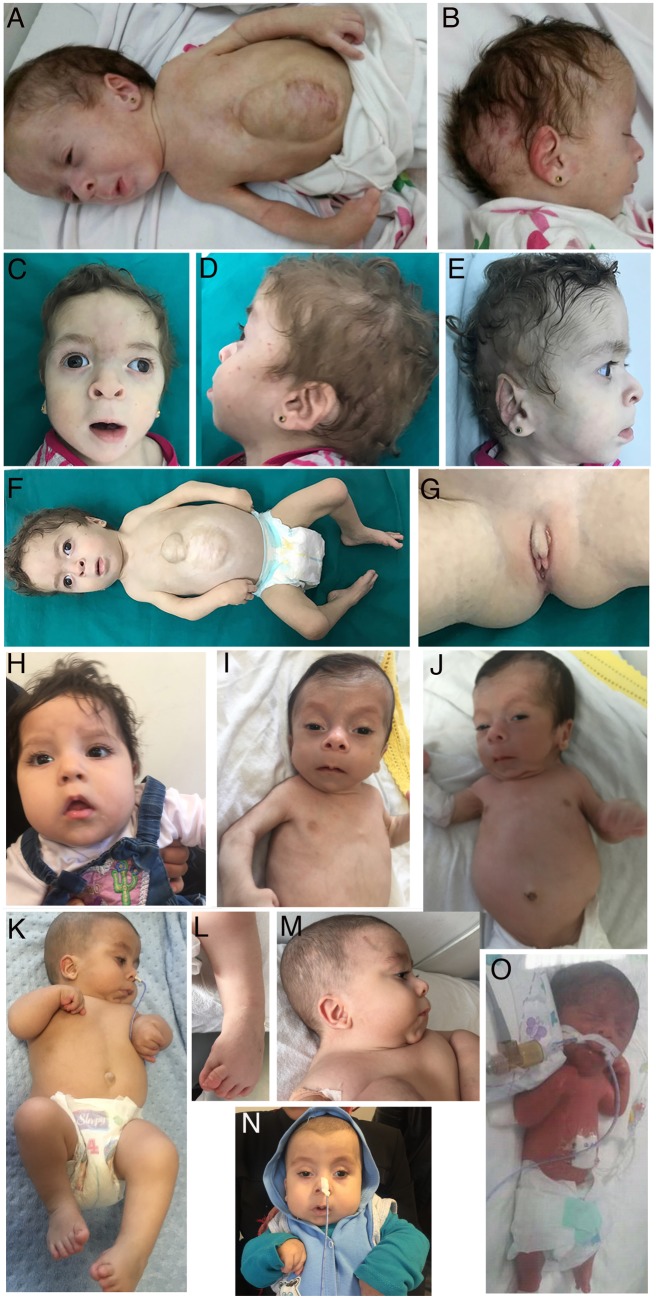
**Clinical features of the patients.** (**A**–**G**) Case 4 (Patient B-IV:4). (**A**) Total view of the proband at the age of 9 months, showing multiple contractures at different joints, and corrected omphalocele. (**B**) Haemangiomas at the right ear and scalp. (**C**) Dysmorphic facial features of the index patient, at the age of 2 years. Note hypertelorism, downslanting palpebral fissure, broad nasal bridge with anteverted nares, smooth philtrum and thin upper lip vermilion. (**D** and **E**) Lateral view of the patient. Note flat facies, microretrognathia and abnormal asymmetric shaped ears. (**F**) Total view of the proband showing multiple contractures at different joints, right talipes equinovarus (TEV) and corrected omphalocele with incisional hernia. (**G**) Abnormal external genitalia with hyperplastic clitoris and hypoplasia of both labia majora and minora. (**H**) Frontal view of Case 7 (Patient D-V:2) showing low set ears, broad nasal bridge, mild hypertelorism and thin upper lip. (**I**–**O**) Cases 9 and 10 (Patients E-III:2 and E-III:1). Photographs of Patient E-III:2 at the age of 3.5 months (**I** and **J**) and 15 months (**K**–**N**). (**I**, **J**, **M** and **N**) Note facial dysmorphism (high arched eyebrows, up-slanting palpebral fissures, hypertelorism, depressed nasal bridge, anteverted nares, low set ears, microretrognathia, long philtrum, sparse eyebrows and hair). (**I**–**J**) Cervical and axillar web. (**J**) Abdominal distension. (**K**–**N**) Flexion contractures of extremities and (**L**) pes equinovarus deformity. (**O**) Case 10 (Patient E-III:1). Note prematurity (born at 29 weeks age of gestation), hypertelorism, macrocephaly, narrow thorax and short limbs.

Case reports are summarized in Table 1, and extensively described below. All patients were born to consanguineous parents, either first or second cousins (Fig. 1). At the time of report, four affected individuals had died. One of these four patients (Case 10, Patient E-III:1) died at the age of 9 days after a preterm birth at 29 weeks, before full phenotypic development could be evaluated. One patient (Case 1, Patient A-III:1) was only first examined at the age of 6 months. The remaining patients all had neonatal developmental and epileptic encephalopathies (DEE) with seizure onset in the first months of life. No ictal EEG recordings were available for re-evaluation in the context of this study. Therefore, seizure classification was based on description of seizures in medical records. Accordingly, seizure types at onset mainly included myoclonic seizures (*n = *5), or epileptic spasms (*n = *6). Both siblings of Family B presented with epileptic spasms and ‘eyelid twitches’. Potentially the latter were myoclonic seizures too. EEG at onset showed suppression-burst or variant with interburst attenuation pattern (Case 8, Patient D-V:3) in seven of eight patients who had recordings during the neonatal period, and hypsarrhythmia in two patients who only had a first EEG taken later in disease course. Seizures were eventually controlled by anti-epileptic drugs in six patients, and were controlled for long periods after addition of vigabatrin in five of them. Head circumference was in the normal range for all patients but one. All living patients had severe psychomotor delay. Brain MRI, when performed, was normal in five patients. One patient presented with hypoplastic corpus callosum and mild-to-moderate dilated subarachnoid spaces, predominating on left hemisphere and mild enlarged lateral ventricles (Case 3, Patient B-IV:4). One patient had a posterior cervical junction (Case 8, Patient D-V:3). Another had an atlanto-axial anomaly with minimal hydrocephalus at 2 months of age (Case 9, Patient E-III:2). In one patient, MRI was normal at the age of 4 months and showed mild cerebral atrophy with ventricular enlargement at 6 years of age (Case 11, Patient F-IV:1). Interestingly, in addition to neurological features, seven patients presented a cleft palate, six joint contractures and five pes equinovarus. Scoliosis was reported in four patients. Both patients from Family B presented omphalocele, and three patients were presenting facial dysmorphic features without clear common elements (Fig. 2).

**Table 1:** Clinical features of the patients with *GAD1* variants

	**Family** A		**Family** B		**Family** C		**Family** D		**Family** E		**Family **F
Case	1 (A-III:1)	2 (A-III:2)	3 (B-IV:4)	4 (B-IV:1)	5 (C-III:1)	6 (C-III:2)	7 (D-V:2)	8 (D-V:3)	9 (E-III:2)	10 (E-III:1)	11 (F-IV:1)
Sex	Male	Female	Female	Male	Female	Male	Male	Female	Male	Male	Female
Age at study	6 y	2 y	Deceased at 2 y	Deceased at 4 y	12 y	Deceased at 2 y	11y	15m	16m	Deceased at 9 d	6 y 11 m
*GAD1* variant	c.1414-1G>C		c.695_697delAGA		c.812_816delTTAAG		c.1591C>T		c.1591C>T		c.1525G>A
GAD67 variant	p.(?)		p.(Lys231del)		p.(Val271Aspfs*9)		p.(Arg531*)		p.(Arg531*)		p.(Glu509Lys)
Age at seizure onset	<6 m	1 d	2 w	2 w	2 w	First days of life	1 d	7 d	1 m	1 d	7 d
Seizure type at onset	ES	ES	ES, ‘eye twitches’	‘eye twitches’, ES	Myo	Myo, T and GTCS	Myo	ES, Myo	ES	Myo	Myo
Evolution of seizures	GTCS after the age of 3 y	Seizure-free from age 3m	Seizure-free from age 9m	Seizure-free from age 2 y	GTCS from the age of 5 m	Increasing seizure frequency (type unknown)	Last seizure at age 10 y	No seizures reported for 2 m	Siezure-free from age 2 m	Deceased at 9th day of life	Tonic, ES, and focal
EEG at onset	HS	S-B	S-B	S-B	S-B	S-B	Dysrhythmia	S-B/burst attenuation	HS	NA	S-B
Drug-resistance	Yes	No	No	No	Yes	Yes	Last seizure at age 10 y	No	No	NA	Occasional seizure
Epilepsy syndrome	WS at first evaluation	Neonatal DEE with S-B	Neonatal DEE with S-B	Neonatal DEE with S-B	Neonatal DEE with S-B	Neonatal DEE with S-B	Neonatal DEE	Neonatal DEE with S-B	WS	NA	Neonatal DEE with S-B
Other neurological features	Axial hypotonia, spasticity, scoliosis	Axial hypotonia, increased muscle tonus limbs Abnormal eye movements	Spasticity, scoliosis	Hyperreflexia, spasticity	Tetraparesis, increased muscle tonus limbs. Conductive hearing loss	Tetraparesis, increased muscle tonus limbs	Axial hypotonia, spasticity, dystonia	Axial hypotonia, mild dystonia	Axial hypotonia, spasticity	NA	Dystonia and hyperkinetic movements
Degree of ID	Profound	Profound	Profound	Profound	Profound	Profound	Profound	Profound	Profound	NA	Profound
Pes equinovarus	No	No	Yes	Yes	Yes	No	No	No	Yes	Yes	No
Omphalocele	No	No	Yes	Yes	No	No	No	No	No	No	No
Cleft palate	Yes	Yes	Yes	No	Yes	Yes	No	No	Yes	Yes	No
Joint contractures	Yes	Yes	Yes	Yes	No	No	No	No	Yes	No	Yes
Dysmorphic facial features	No	No	Yes	Yes	No	No	No	No	Yes	No	No
Brain MRI (age)	Nl (2.5 y)	NA	Mild-to-moderate cerebral and cerebellar (progressive) atrophy L > R, hypoplastic CC (1 m) and (2 y) and cervical notch	NA	Nl (3 y)	Nl (1 y)	Nl	Posterior cervical junction notch	MRI Nl (50 d); CT atlanto-axial anomaly, minimal hydrocephalus (2 m)	Cranial ultrasound: germinal matrix haemorrhage	Nl (4 m) / mild cerebral atrophy (13 m) / mild cerebral atrophy with ventricular dilation (6 y)

*GAD1* transcript NM_000817.3

B6 = vitamin B6; CC = corpus callosum; CLB = clobazam; CLZ = clonazepam; d = days; DEE = developmental and epileptic encephalopathy; DZP = diazepam; ES = epileptic spasms; GTCS = generalized tonic-clonic seizures; HS = hypsarrhythmia; ID = intellectual disability; L = left; LEV = levetiracetam; LTG = lamotrigine; m = months; Myo = myoclonic seizures; NA = not ascertained; Nl = normal; NZP = nitrazepam; PB = phenobarbital; PRM = primidone; R = right; S-B = suppression-burst pattern; T = tonic seizures; VGB = vigabatrin; VPA = valproic acid; w = weeks; WS = West syndrome; y = years.

### Case reports

#### Family A

##### Case 1: Patient A-III:1

Case 1 was a 6-year-old male, the first child of healthy first cousin parents of Algerian origin (Fig. 1A). There was no family history of epilepsy, intellectual disability or other neurodevelopmental disorders. The patient was born at term (40 weeks of gestation). Birth weight was 3.660 kg (mean), height was 48 cm [−1 standard deviation (SD)] and head circumference 34 cm (−1 SD). Examination at birth showed cleft soft palate with surgical correction at 2 months.

**Figure 3:**
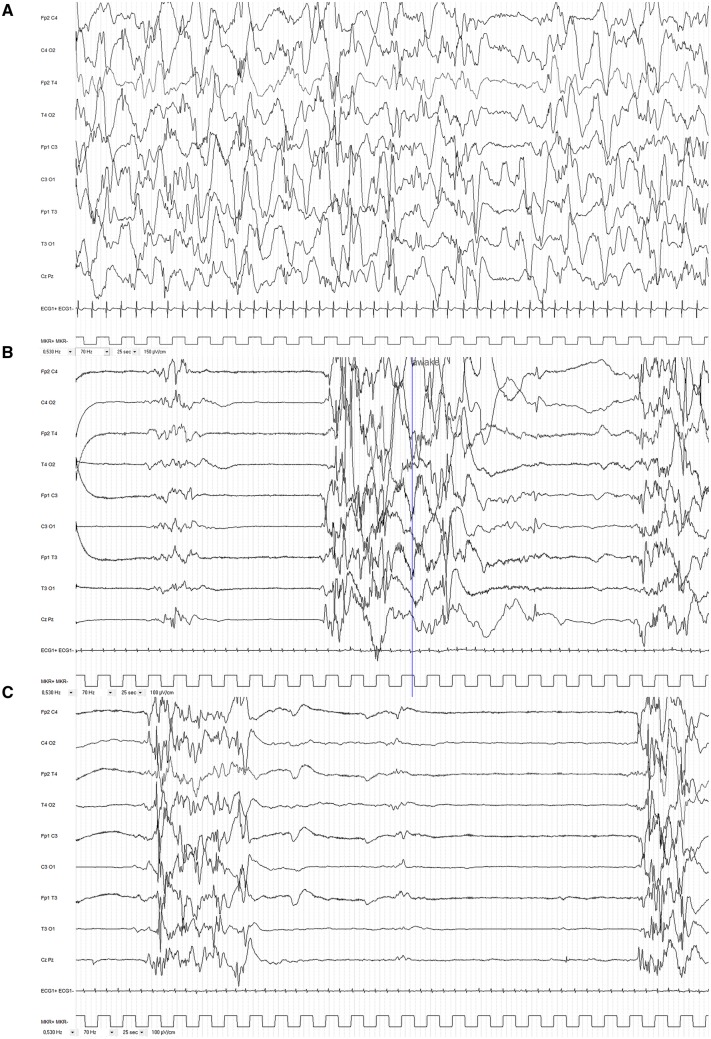
**Interictal EEGs.** (**A**) Case 1 (Patient A-III:1). Hypsarrhythmia at the age of 5 months; note random high amplitude slow waves and spikes during sleep with episodes of voltage attenuation. (**B** and **C**). Case 2 (Patient A-III:2). (**B**) Suppression-burst pattern (S-B) persists at the age of 5 weeks (42 weeks age of gestation). Note bursts of high-voltage slow waves intermixed with high amplitude spikes, polyspikes and fast rhythms, lasting ∼4–5 s and alternating with complete suppression lasting ∼4–5 s in wakefulness. Some bursts could terminate with slow waves mixed up with rare sharp waves predominating over central regions. Note also brief and rare bursts of moderate amplitude theta/delta waves. (**C**) Longer interburst interval (8–10 s) in sleep.

When first referred to the neuropaediatric unit, at 5 months of age, the patient had developmental regression with loss of visual contact, abnormal neurological examination with opisthotonous and axial hypotonia. Weight and height were at mean and head circumference at +2 SD. EEG showed hypsarrhythmia with brief episodes of voltage attenuation without clear clinical correlate (Fig. 3A). Brain MRI was normal. Metabolic screening was normal in blood, urine and CSF (including amino acid chromatography, organic acid chromatography, acylcarnitine, lactate and pyruvate, neurotransmitters). The child was treated with vigabatrin and was lost to follow-up and rehabilitations for the following 2 years.

At 2 years and 6 months of age, he presented with spastic quadriparesis, right plagiocephaly, poor visual contact, but had the ability to smile. EEG still showed hypsarrhythmia with clinically subtle spasms. Brain MRI, including spectroscopy, was normal.

At last follow-up, at 6 years of age, the patient was not able to sit unassisted, had spastic tetraparesis with limited extension of knees and elbows, and showed no verbal communication. The last EEG showed low-voltage and slow background activity with multifocal low amplitude spikes. No seizures were recorded.

##### Case 2: Patient A-III:2

Case 2 was a 2.5-year-old female, second affected offspring in Family A, who was born at 37 weeks of gestation (Fig. 1A). Birth weight was 3.190 kg (mean), height was 48 cm (mean) and head circumference 34.7 cm (mean). She had a cleft soft palate. The patient was admitted at 2 days of age to neonatal intensive care unit due to episodes with eye revulsion, cyanosis and spasms-like movements. EEG showed a suppression-burst pattern (Fig. 3B and C). She was treated with levetiracetam and vitamins (B1, B6 and biotin). Vigabatrin was added at 1 month because of persistence of spasms. The combination of levetiracetam and vigabatrin lead to seizure control at the age of 3 months. She had surgical correction of cleft soft palate at 10 months of age. At last follow-up at 26 months of age, she had severe developmental delay with axial hypotonia and absence of head control, segmental hypertonia with limited extension of knees and, no visual contact with abnormal eye movements and no verbal communication. EEG showed normal background activity without epileptic abnormalities.

#### Family B

##### Case 3: Patient B-IV:4

Case 3 was a female, the third child of apparently healthy first-cousin parents of Egyptian origin (Fig. 1B). She was a product of *in vitro* fertilization (IVF) and pregnancy was complicated by polyhydramnios. Prenatally detailed scan revealed a liver containing omphalocele, intestinal atresia and right talipes equinovarus. Delivery was at term by caesarean section, and birthweight was 2.900 kg. Examination at birth showed omphalocele and cleft soft palate. A subcutaneous branchial fistula was noted at the left side of the neck that spontaneously obliterated before 6 months of age (Fig. 2B). Resection anastomosis was performed at 2 days of age for correction of omphalocele and jejunal atresia (Fig. 2). The patient was admitted to the neonatal intensive care unit for 2 weeks. Seizures were first noted at 2 weeks of age in the form of eye twitches and epileptic spasms. She was treated with phenobarbital, valproic acid, oxcarbazepine, and eventually became seizure-free with vigabatrin at the age of 9 months. At follow-up at 9 months of age, the patient was severely developmentally delayed with no head support. At 2 years of age, she could not sit independently and was unable to talk.

The patient presented with multiple congenital anomalies and dysmorphias: plagiocephaly with bitemporal narrowing and a forehead naevus flammeus, frontal upsweep and facial hirsutism were noted. Further, she showed mid-face hypoplasia, facial asymmetry, and microretrognathia. The eyes had bluish sclera, large cornea, squint and hypertelorism were present, as well as wide downslanting palpebral fissure and infraorbital creases. Eyebrows were broad and flared. She had broad depressed nasal bridge, broad nasal ridge and tip with enlarged anteverted nares, wide nasal base in addition to smooth long philtrum. She had abnormal shaped ears with low setting, over folding of the helix, prominent inferior crus of antihelix, serpiginous and prominent stem of antihelix, prominent antitragus and narrow external auditory meatus. The ears were asymmetric with the serpiginous prominent helix crus in the left ear and crumpled helix in the right. The oral region showed absent cupid’s bow with a thin upper lip vermilion, thick lower lip vermilion, underdeveloped nasolabial fold and limited opening of mouth cavity. Delayed teeth eruption was noted with only two lower central incisors. The palate was high arched with small cleft in the soft palate (Fig. 2). Multiple skin hemangiomas were noted at the right eyelid, right ear and scalp. She had a short neck, with anterior webbing. The shoulders were sloping and narrow, and axillary webbing was noted. Limbs exhibited a limited range of movement in all joints, with limited extension of elbows and knees, talipes equinovarus or clubfoot of the right foot, ulnar deviation at the wrist and skin dimples at the dorsal aspects of both elbows and wrists (Fig. 2). She had small hands. All fingers were broad with camptodactyly, short distal phalanges, prominent digital pads, and shallow/absent flexion creases in plantar and dorsal surfaces. Thumbs were adducted bilaterally. Both feet had rocker bottom appearance. The thorax was short with hypoplastic widely spaced nipples and corrected omphalocele was noted at the anterior abdominal wall with incisional hernia. She also had scoliosis. Examination of the genitalia revealed large clitoris with hypoplastic labia minora and majora (Fig. 2). Head circumference was within the normal range, while the weight and length were far below normal values for her age (<3rd centile). The patient died at the age of 2 years, from failure to thrive and respiratory infection.

**Figure 4:**
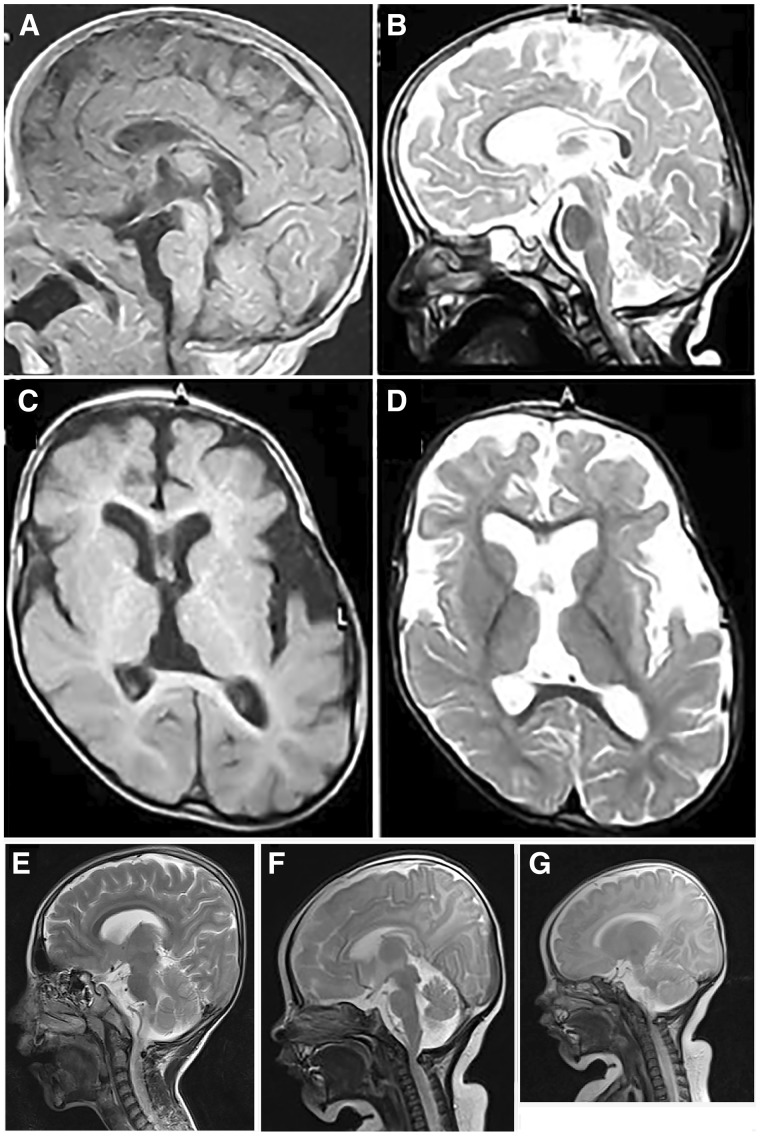
**Brain MRI.** (**A**–**D**) Case 3 (Patient B-IV:4). Sagittal T_1_ and T_2_ cuts through the midline at the age of 1 month (**A**) and 2 years (**B**) showing hypoplastic corpus callosum, mainly body and to a lesser degree its genu, progressive cerebellar and cerebral atrophy and a cervical notch. Axial cuts T_1_ (**C**) and T_2_ (**D**) at the age of 2 years demonstrating mild-to-moderate cortical atrophy predominating on left hemisphere and mildly enlarged lateral ventricles. (**E**–**G**) Cases 7 and 8 (Patients D-V:2 and D-V:3). (**E**) Sagittal MRI image of Case 7 (Patient D-V:2) showing prominent inner liquor spaces without overt brain malformations. (**F**) Sagittal MRI image of Case 8 (Patient D-V:3) showing prominent inner liquor spaces without apparent brain malformations except a cervical notch causing an impression in the upper cervical spinal cord area. (**G**) Normal cerebellar size is demonstrated in Case 8 (Patient D-V: 3).

EEG showed a suppression-burst pattern at onset, evolving to hypsarrhythmia on recording at the age of 6 months. Brain MRI at 1 month of age and at the age of 2 years revealed hypoplastic corpus callosum, mainly of the body and to a lesser degree of the genu and dilated subarachnoid spaces predominating on left hemisphere and mildly enlarged lateral ventricles (Fig. 4A–D). Ultrasonography of the abdomen and pelvis was unremarkable and karyotype was 46, XX.

##### Case 4: Patient B-IV:1

Case 4 was a similarly affected brother, who passed away at the age of 4 years due to pulmonary infection and severe growth retardation. He was delivered by elective caesarean section at term with average birth weight (50th centile). At birth, omphalocele and bilateral talipes equinovarus were observed. He was admitted to neonatal intensive care unit for 3 weeks due to pulmonary infection, followed by surgical repair of the omphalocele. He also had failure to thrive, seizures, arthrogryposis and global developmental delay. Seizures were first noted at 2 weeks of age in the form of eye twitches and epileptic spasms. He was treated with phenobarbital, valproic acid and eventually became seizure-free with vigabatrin at the age of 2 years. He presented multiple congenital anomalies and dysmorphias similar to his sister. His body weight at 4 years of age was 5 kg (far below 3rd centile).

#### Family C

##### Case 5: Patient C-III:1

Case 5 was a 12-year-old female, the first child of an apparently healthy first-cousin Turkish couple (Fig. 1C). She had an onset of eyelid-myoclonia at the age of 2 weeks, and 1 week later also showed myoclonic seizures of the upper limbs. At the age of 5 months, she developed generalized tonic-clonic seizures. The patient was initially treated with vitamin B6 and B12, and later with topiramate, phenobarbital, levetiracetam, zonisamide, valproate, azetazolamide, lamotrigine and vigabatrine without any effect of seizure frequency. Currently, she suffers from a refractory epilepsy with bursts of myoclonic seizures (once a month) and tonic-clonic seizures (once a week) despite treatment with levetiracetam and diazepam. Initial EEG in the second week of life showed a suppression-burst pattern. Later EEGs showed multifocal spike wave complexes with a generalized and focal slowing with shifting maxima. In the EEG at the age of 7 years, photosensitivity was observed at frequencies from 1 to 6 Hz in the form of generalized sharp waves, and at 7 to 10 Hz in the form of occipital sharp wave. Brain MRI at the age of 3 years and 1 month was normal, as was neurometabolic screening. She developed a profound intellectual disability, with absence of expressive and receptive speech, absence of head control, and no fixation of objects. First available neurological exam at the age of 5 months showed a generalized hypotonia with some limited movements of all limbs. At the age of 2 years there was a regression evolving to a complete tetraparesis, with generalized increased muscle tonus without clear pyramidal signs, and pes equinus. She has a palatine cleft, a talipes on the left side and a conductive hearing loss. She suffers from recurrent fever episodes without any signs of infection. No further facial dysmorphic signs were observed.

##### Case 6: Patient C-III:2

Case 6 was the second child of Family C (Fig. 1C). He showed a similar phenotype as his older sister (Patient C-III:1), with refractory epilepsy with myoclonic, tonic and tonic-clonic seizures, from the first day of life. As his sister, he had a palatine cleft. Neonatal EEGs showed a suppression-burst pattern and brain MRI was normal. Neurometabolic screening was normal. Seizures deteriorated under ketogenic diet and he died from refractory seizures at the age of 2 years. At that time, he had a profound intellectual disability with tetraparesis with increased muscle tonus.

There was a familial history of seizures, as the paternal grandfather (Subject C-I:1) had two tonic-clonic seizures at the age of 40 and 47 years, and the maternal grandfather (Subject C-I:3) had tonic-clonic seizures arising from sleep since the age of 40 years. There was no information on EEG or MRI available.

#### Family D

##### Case 7: Patient D-V:2

Case 7 was a 10-year-old male, the second child of healthy second-cousin parents from Iran with a family history of epilepsy and development delay (Figs 1D and 2J). He was delivered at 37 weeks of gestation by caesarean section. First myoclonic seizures were noted within 1 day after birth and EEG showed dysrhythmia. The seizures evolved into epileptic spasms. The patient was treated with clonazepam, primidone and vitamin B6 under which he remained seizure-free from the age of 10 years until the most recent follow-up examination at 11 years of age. His neurodevelopment was profoundly delayed with no ability to walk. However, brain MRI indicated prominent inner liquor spaces without overt brain malformations (Fig. 4E). He initially showed reduced muscle tone and developed spasticity and dystonia as well as scoliosis later during the disease course.

##### Case 8: Patient D-V:3

Case 8 was a 2-month-old female, the third child of Family D (Fig. 1D), delivered at 38 weeks of gestation by caesarean section. She was noted to suffer from epileptic spasms and myoclonic seizures at 7 days of age. EEG showed burst attenuation initially and scattered sharp waves later during the disease course. Nitrazepam, phenorbarbital and vitamin B6 resulted in a seizure-free episode of 2 months. At follow-up at 15 months of age, she was able to roll over onto her sides but was unable to sit unsupported. Head circumference at 15 months of age was 44 cm (1.2 cm <3rd centile). Brain MRI at the age of 10 years did not reveal any structural defects except a cervical junction notch with impression on the spinal cord (Fig. 4F and G).

#### Family E

##### Case 9: Patient E-III:2

Case 9, the proband from Family E, was the second child of first-cousin parents. He was referred to Istanbul University Cerrahpasa Medical Faculty, Pediatric Genetic Department at 2 months of age due to facial dysmorphism, development delay, cleft palate, Hirschsprung disease, and pes equinovarus deformity (Fig. 2). Intrauterine hydrocephalus was diagnosed in the third trimester of pregnancy. The patient was delivered at 37 weeks of gestation by caesarean section. The birth weight and length were 3.410 kg and 45 cm, respectively. He was followed-up in the neonatal intensive care unit for 27 days because of respiratory distress, cleft palate and abdominal distension. Intestinal obstruction was suspected during neonatal intensive care unit follow-up. Double contrast barium enema demonstrated colonic dilatation. The diagnosis of Hirschsprung was confirmed by rectal biopsy.

Initial physical examination of the proband revealed facial dysmorphism (high arched eyebrows, upslanting palpebral fissures, hypertelorism, depressed nasal bridge, anteverted nares, long philtrum, thin lips, low-set ears, auricular pit, microretrognathia), cleft palate, cervical and axillar webbing, flexion contractures of elbows and knees, abdominal distension, rectus diastasis, umbilical hernia, 3-cm palpable liver below costal margin, hirsutism of back, hypotonia and pes equinovarus deformity (Fig. 2). Epileptic spasms were observed during physical examination. Retrospectively these were present from 1 month of age, but were not recognized as seizures by parents. EEG revealed hypsarrhythmia. Phenobarbital, vigabatrin and vitamin B6 were started as initial treatment. Brain CT scan at 2 months of age demonstrated minimal hydrocephalus and atlantoaxial anomaly. Although epileptic spasms were well controlled with antiepileptic treatment, hypsarrhythmia was still present in the control EEG, at 6 months of age. On eye examination, nystagmus, alternating esotropia, and bilateral pale optics disk were observed. Audiogram was normal at birth, but he failed the hearing test performed at 1 year of age.

In the follow-up, the patient had severe recurrent aspiration pneumonia requiring hospitalization and oxygen treatment. Chest CT revealed bilateral atelectasis, fibrotic changes and decrease in right lung volume, due to the intrathoracic placement of the right liver lobe. Evaluation for diaphragmatic hernia was normal. Barium oesophagogram showed nasal regurgitation and laryngotracheal aspiration, requiring nasogastric tube feeding.

Echocardiography showed a small patent foramen ovale, small atrial septum defect (both resolved in control echocardiography), focal septal hypertrophy, and urinary ultrasonography revealed grade 1 nephrocalcinosis.

At last examination, at 15 months of age, the patient did not have head control or eye contact. His weight, length, and head circumference were 8.800 kg (3–10th centile), 67 cm (<3rd centile) and 48.5 cm (50–97th centile), respectively. He showed a large anterior fontanelle (4 × 3 cm), sparse hair and eyebrows, and 5 cm palpable liver below the costal margin. He had axial hypotonia and spasticity of the limbs. Persistence of flexion contractures of knees and elbows, and ulnar deviation of the wrist were observed. The hirsutism of the back was no more observed at the last examination.

##### Case 10: Patient E-III:1

Case 10 (Patient E-III:1), the first child of the parents of proband Case 9, was born at 29 weeks of gestation, had cleft palate, short neck, narrow thorax, myoclonic seizures, congenital heart disease (large patent ductus arteriosus, small atrial septum defect moderate pulmonary hypertension), abdominal distension and pes equinovarus deformity similar to the proband, and died at postnatal Day 9 due to sepsis (Fig. 2).

#### Family F

##### Case 11: Patient F-IV:1

Case 11 was a 7-year-old female, the first child of healthy first-cousin Brazilian parents of Caucasian and African ancestry. There was no family history of any developmental disorder or epilepsy. Pregnancy was uneventful and she was delivered after 37 weeks of gestation by caesarean section. Her physical examination was normal at birth and she was discharged at 3 days of age, apparently in good health condition.

At 7 days of age, the family recognized abnormal fast facial twitches with blinking followed by staring. She was evaluated by a child neurologist and was treated initially with phenobarbital and 1 month later with valproic acid, without success. EEG was abnormal with multifocal discharges and periods of suppression-burst. Epilepsy remained uncontrolled, with several daily seizures (focal, epileptic spasms and star gazing). At 10 months of age, clobazam was introduced as an add-on drug without improvement, as she still had daily seizures. Finally, at 11 months of age she started vigabatrin (max dose of 1000 mg), which completely controlled the seizures after 2 weeks later. Vigabatrin was continued for 2 years. The patient is currently receiving lamotrigine (100 mg/day), levetiracetam (750 mg/day), and clobazam (10 mg/day), with the occasional seizure. Several follow-up EEGs demonstrated multifocal epileptiform activity, sometimes more intense in the centrotemporal region. Additional tests included two brain MRIs, showing mild cerebral atrophy at 13 months of age with ventricular dilation at the age of 6 years. Metabolic work-up was normal. She has been in a continuous rehabilitation programme, which includes physical, occupational and speech therapy, from the age of 3 months.

At last examination, at 6 years of age, her weight was 17 kg, occipital frontal circumference 50 cm with positional plagiocephaly, inverted nipples, hirsutism and severe scoliosis. No dysmorphic facial features were seen. Neurological examination disclosed a profound developmental delay/intellectual disability. She had good eye contact and showed interest in watching cartoons and turned her face to sounds. She had incomplete head support. She was able to roll over on her back but could not sit, even with support. The patient did not follow commands or speak a single meaningful word. She kept her hands fisted with flexion of wrists and her arms movements were uncoordinated and choreathetotic. She could not grasp objects. Muscle tonus in the upper limbs was variable with hypo- or hypertonia and her lower limbs were spastic with brisk reflexes and Babinski sign.

### Genetic findings

Familial genotyping results and identified *GAD1* variants are summarized in Fig. 1 and Supplementary Fig. 1. After stringent filtering of WES or WGS data, rare homozygous bi-allelic variants in *GAD1* were retained as the only likely pathogenic variant in all affected individuals (Supplementary Fig. 1). In all families, Sanger sequencing confirmed segregation of the *GAD1* variants with the disease phenotype (Supplementary Fig. 1) and a recessive mode of inheritance associated with the disease. Detailed genetic results are provided in the Supplementary material.

Four of six families carried a homozygous variant predicted to truncate the protein (Families A, C, D and E). The c.1414-1G>C variant in Family A was predicted to abolish the acceptor splice-site of *GAD1* exon 15 by *in silico* splicing programmes (MaxEntScan and GeneSplicer). cDNA analysis confirmed the absence of normal mRNA product with three aberrant isoforms: one with exon 15 skipped, one with 76 bases of intron 14 retained and one with the first seven bases of exon 15 deleted.

In Family C, a homozygous 5-bp deletion, c.812_816delTTAAG, leading to a frameshift p.(Val271Aspfs*9) was identified **(**Fig. 1C and Supplementary Fig. 1C). Families D and E, of Persian and Turkish origin, respectively, shared the same *GAD1* c.1591C>T variant leading to a premature stop codon p.(Arg531*) **(**Fig. 1D and E and Supplementary Fig. 1D and E). Given the relative geographical proximity, a common haplotype is possible.

The proband of Family B carried a novel homozygous 3-bp deletion in exon 7, c.695_697delAGA (Fig. 1B and Supplementary Fig. 1B), leading to the deletion of a highly conserved lysine at position 232, p.(Lys232del) (Supplementary Fig. 2A). The variant is not present in any public databases [1000 Genome Project Database, Greater Middle East (GME), ExAC/GnomAD browsers]. *In silico* prediction programme predicts the p.(Lys232del) variant to be disease-causing (MutationTaster). In addition, SpliceAid2 predicts that the 3-bp AGA at position c.695_697 of *GAD1* mRNA is part of consensus binding sequences for seven different splicing factors. Consequently, c.695_697delAGA is predicted to result in a splice site loss, leading to a frameshift and subsequently to a premature termination codon. Because of the lack of autopsy material from the deceased affected siblings, *in vitro* protein expression of wild-type and mutated GAD67 was studied, using an *Escherichia coli* cell-free expression system. Western blotting of wild-type and p.Lys232del-GAD67, however, revealed that p.Lys232del-GAD67 was expressed at a comparable level as wild-type-GAD67, which is not in favour of an effect on splicing (Supplementary Fig. 3).

In Family F, another homozygous missense variant was identified, p.(Glu509Lys) (Supplementary Fig. 1F), which is absent from the gnomAD database. It is predicted pathogenic by DANN, GERP, LRT, MutationAssessor, MutationTaster and PROVEAN prediction scores as it affects an evolutionarily extremely well-conserved residue (Supplementary Fig. 2B) within the pyridoxal-dependent decarboxylase conserved domain, indicating that the variant may damage protein function. The HOPE bioinformatics resources were applied to generate a GAD67 structural model and to predict the effects of the missense allele (Supplementary Fig. 4). The wild-type glutamate residue is predicted to form salt bridges with the arginine at position 316, the lysine at position 318 and the lysine at position 389. These ionic interactions will likely be disturbed in the mutated protein as the wild-type amino acid glutamate is negatively charged, while the mutated amino acid, lysine, is positively charged with a larger side chain. Further reversal of charge further may cause repulsion between residues in the protein core and because of the larger side chain, the mutated amino acid is predicted to no longer fit into the domain core.

## Discussion

We describe 11 patients, from six independent families, presenting early-onset DEE and a variable association of cleft palate, omphalocele, equinovarus and/or more widely distributed joint contractures, caused by bi-allelic variants of *GAD1*. Four of six families carried a homozygous variant leading to a truncated protein (Supplementary Fig. 5), supportive of a complete loss-of-function mechanism. *GAD1* constraint scores calculated on the gnomAD dataset are also in line with an autosomal recessive inheritance of loss-of-function variants, as the pRec score (Lek *et al.*, 2016) (probability of being intolerant to bi-allelic loss-of-function variants) is 0.934, above the 0.9 threshold to consider a gene intolerant to bi-allelic variants.

In addition to nonsense and frameshift variants, we found one deletion of a highly conserved lysine [c.695_697delAGA, p.(Lys232del), in Family B]. *In silico* predictions were also in favour of a splicing effect. We could not prove an effect of this in-frame deletion on the expression level of GAD67. However, the normal result of the cell-free expression assay (used in the absence of tissue of the deceased siblings) does not exclude a potentially deleterious impact of the variant on pre-mRNA splicing, as suggested by *in silico* prediction programmes, as the assay begins from mature *GAD1* cDNA sequence, bypassing splicing events. The recapitulation of the knockout mouse model phenotype (co-occurrence of cleft palate and omphalocele) in this family, together with phenotypic similarities with the remaining families, is strongly supportive of pathogenicity of this variant, while loss of function could not be formally demonstrated.

A remarkably increased muscle tone was a core clinical feature seen in all patients. An increase in muscle tone is also the hallmark feature of stiff person syndrome, a disorder linked to anti-GAD antibodies. This autoimmune disorder is thought to result in an impairment of spinal and supra-spinal inhibitory neurons, leading to excessive excitatory drive upon the motor neurons. Whereas in stiff person syndrome axial muscles are predominantly affected, patients reported in our manuscript often had axial hypotonia. This most likely reflects the detrimental impact of a constitutive GABA deficiency on neurodevelopment. Indeed, given the pivotal role of GABA as an inhibitory neurotransmitter and its role in the development of the nervous system, the association of *GAD1* loss-of-function mutations with DEE is not unexpected. *De novo* mutations in several GABA receptor genes have previously been described to lead to DEE (Epi4K Consortium *et al.*, 2013; Hamdan *et al.*, 2017; Moller *et al.*, 2017). Notably, loss-of-function variants in *GABRA3*, encoding the α_3_-subunit of the GABA_A_ receptor, have been associated with a DEE including dysmorphic features and a cleft lip (Niturad *et al.*, 2017). In addition, GABA appears to be essential for foetal movement, as well as developmental processes that depend on foetal movements (Saito *et al.*, 2010; Kakizaki *et al.*, 2015). Several studies demonstrated that both the cleft palate and the omphalocele seen in *Gad1^−/−^* mice are the consequence of reduced foetal movements (Laprade and Soghomonian, 1995; Iseki *et al.*, 2007; Saito *et al.*, 2010; Kakizaki *et al.*, 2015). Further studies demonstrated that the presence of cleft palate is due to a defect of foetal oral movement of the tongue during palatal development and *Gad1* is specifically required in the CNS for normal palatal formation (Iseki *et al.*, 2007; Oh *et al.*, 2010). Furthermore, it has been suggested that lack of tongue movement may result in a tongue position enhancing cleft formation, such as tongue positioning in-between the two palate shelves, as observed in a recent prenatal MRI study for Robin syndrome patients with clefts (Resnick *et al.*, 2018). Likewise, it has been suggested that movement reduction leads to a hunched posture in the foetus and increased intra-abdominal pressure, resulting in failure of retracting umbilical hernia and omphalocele (Saito *et al.*, 2010; Kakizaki *et al.*, 2015). Also the pes equinovarus, joint contractures and arthrogryposis, seen in several of the patients in our study, are known to reflect a reduction of foetal movements (Hall, 1997). As such, the non-neural phenotypes seen in our cohort of patients should probably be seen as secondary to the severe CNS involvement.

Despite identical mutation in Families D and E, there was some phenotypic variability between both families; in particular there was an absence of pes equinovarus, joint contractures and cleft lip in Family D. This might be influenced by variability in genetic backgrounds or environmental factors that modulate movement *in utero*. The cervical notch observed in the brain MRI of the proband of Family D is likely unrelated to the *GAD1* variant as no other cases in our cohort displayed a similar finding. Also, in Family C, both paternal and maternal grandfather had late onset epilepsy. Epilepsy type and disease course were, however, totally different from probands, and we thus do not assume that they are caused by the same genetic defect. However, we cannot exclude that other epilepsy susceptibility variants segregate in the family and possibly might influence the probands’ phenotype.

Finally, a remarkably good response of seizures to vigabatrin was seen in five of seven individuals who were treated with the drug. Vigabatrin inhibits the GABA transaminase, responsible for GABA catabolism, thus increasing the synaptic concentration of GABA reduced by *GAD1* variants. It should be noted however, that this good response seems to be driven by seizure semiology, as particularly patients with epileptic spasms appeared to benefit from vigabatrin. Vigabatrin is the standard treatment choice for epileptic spasms, a seizure type that is known to respond well to this drug regardless of underlying aetiology. Also, given the importance of GABA in prenatal neurodevelopment, it remains to be demonstrated whether postnatal treatment initiation still has an impact on intellectual functioning, and whether this exceeds the expected beneficial effect of a reduction of seizure burden on neurodevelopment. Nevertheless, this observation should be an incentive to validate the role of vigabatrin as a targeted (seizure) treatment for *GAD1*-related disorder. Several examples of the usefulness of ultra-fast genomic sequencing for medical management have been reported (Smith *et al.*, 2015; Friedman *et al.*, 2019). Given the early-onset of seizures and the potential importance of early treatment initiation, we thus strongly advocate early clinical genomic sequencing in patients with early-onset seizures.

In conclusion, our study provides evidence of the essential role of the GAD67 enzyme during human foetal development. Bi-allelic loss-of-function variants in humans fully recapitulate the association of epilepsy, cleft palate and omphalocele observed in animal models. Moreover, we emphasize the importance of early genetic testing to drive therapeutic decisions for DEE.

## Web resources

The following databases were used in this study:

UCSC Human Genome Database: http://www.genome.UCSC.edu

The Exome Variant Server: NHLBI Exome Sequencing Project (ESP), Seattle, WA; URL: http://evs.gs.washington.edu/EVS/

1000 Genome Project Database: http://browser.1000genomes.org/index.html

Human Background Variant DataBase: http://neotek.scilifelab.se/hbvdb/

Greater Middle East (GME) Variome web: http://igm.ucsd.edu/gme/index.php

Genome Aggregation Database (gnomAD): http://gnomad.broadinstitute.org/

Exome Aggregation Consortium (ExAC): http://exac.broadinstitute.org/

Gene Matcher (GeneMatcher): https://genematcher.org/

MutationTaster: http://mutationtaster.org/

MaxEntScan: http://hollywood.mit.edu/burgelab/maxent/Xmaxentscan_scoreseq.html

GeneSplicer: http://www.cbcb.umd.edu/software/GeneSplicer/gene_spl.shtml

SpliceAid2: http://www.introni.it/spliceaid.html

RCSB Protein Data Bank: https://www.rcsb.org/structure/2OKJ

## Supplementary Material

awaa085_Supplementary_Data

## Appendix 1

The AR working group of the EuroEPINOMICS RES Consortium consists of the following consortium members: Ingo Helbig; Sarah von Spiczak, Stephanie Baulac; Nina Barisic; Rudi Balling; Hande Caglayan; Dana Craiu; Renzo Guerrini; Karl Martin Klein; Carla Marini; Hiltrud Muhle; Felix Rosenow; Jose M. Serratosa; Katalin Sterbova; Yvonne Weber.

## Abbreviations

DEE =: developmental and epileptic encephalopathies
