# Supplementary material for: Bi-allelic GAD1 variants cause a neonatal onset syndromic developmental and epileptic encephalopathy
Source: Brain. 2020 Apr 13;143(5):1447–61. doi: 10.1093/brain/awaa085 (PMC7241960; doi:10.1093/brain/awaa085)
Supplement: awaa085_Supplementary_Data [file awaa085_supplementary_data.zip › awaa085-suppl_data/brain-2019-01328-File007.pdf]

Supplementary information

Content

1. **Supplementary Methods**
2. **Supplementary Results**
3. **Supplementary References**
4. **Supplementary Figure legends**

1. **Supplementary Methods**

Genetic analysis

DNA isolation

Extraction of genomic DNA was performed from whole blood from family members, using DNeasy Blood & Tissue kit (Qiagen, Hilden, Germany), according to the manufacturer's instructions.

Array-CGH

Array-CGH was performed for the case A-III:1 from Family A using a 180-k microarray (AMADID: 022060), according to the manufacturer's instructions (Agilent Technologies, Santa Clara, CA, USA). Results were analyzed using Agilent Cytogenomics software v3.3.

Next generation sequencing

After standard DNA extraction, DNAs of the two infants (A-III:1 and A-III:2) from **Family A** were fragmented using sonication (Covaris, Woburn, MA, USA). Library preparation and target enrichment were performed with the MedExome probes (Roche Sequencing Solutions, Madison, WI, USA), following manufacturer's recommendations. Paired-end 2x150 sequencing was performed on a NextSeq500 HighOutput Flowcell using v2 chemistry

(Illumina, San Diego, CA, USA). Genomic alignment on GRCh37 reference and small variant calling were performed respectively using BWA-MEM v0.7 and GATK HaplotypeCaller v3.4. Considering autosomal recessive hypothesis, shared homozygous autosomal variants were kept for the first analysis. Variant classification followed ACMG recommendations (Richards et al., 2015). Relevant variants were confirmed using Sanger sequencing. Whole exome sequencing (WES) was performed on DNA from the proband (B-IV:4) in **Family B**, as previously described (Kariminejad et al., 2016). Briefly, target enrichment was performed with 3 μg genomic DNA using the SureSelectXT Human All Exon Kit version 5 (Agilent Technologies, Santa Clara, CA, USA) to generate barcoded whole-exome sequencing libraries. Libraries were sequenced on the HiSeq2000 platform (Illumina, San Diego, CA, USA) as paired-end 2 ×100-bp reads with 60x coverage. Quality assessment of the sequence reads was performed by generating QC statistics with FastQC (http://www.bioinformatics.bbsrc.ac.uk/projects/fastqc). Read alignment to the reference human genome (hg19, UCSC assembly, February 2009) was done using BWA (Li and Durbin, 2009) with default parameters. After removal of PCR duplicates (Picard tools, http://picard.sourceforge.net) and file conversion (SAMtools) (Li et al., 2009), quality score recalibration, indel realignment and variant calling were performed with the HaplotypeCaller algorithm in the GATK package (McKenna et al., 2010), based on established best practices (DePristo et al., 2011).

Variants were annotated with ANNOVAR (Wang et al., 2010) using a wide range of databases such as dbSNP build 135, dbNSFP, KEGG, the Gene Ontology project and tracks from the UCSC. A filtering strategy, directed to disease gene candidates, was performed by QIAGEN's Ingenuity® Variant Analysis™ software (www.qiagen.com/ingenuity) from QIAGEN Redwood City. Ingenuity Variant Analysis combines analytical tools which annotates variants and displays data including Polyphen, SIFT and CADD scores. We focused

initially on coding variants in genes causing congenital and neurogenetic diseases that lead to a missense change, stop gain or stop loss, frameshift or essential splicing change. Given the known consanguinity in this family, the initial filtering strategy was further also focused on homozygous variants. Only those changes that were predicted to be damaging or with unknown impact were analysed. We excluded variants that were frequent in control datasets (>1% in dbSNP, (Sherry et al., 2001), the Exome Variant Server (EVS) (NHLBI), the 1000 Genome Project Database, the Exome Aggregation Consortium (ExAC) and the Human Background Variant Database.

In **Family C**, WGS was performed on DNA extracted from both healthy parents (C-II:1 and C-II:2) and the affected siblings (C-III:1 and C-III:2) (Complete Genomics, Mountain View, CA, USA). Data was generated, annotated, and variant prioritized, as previously described (Schubert et al., 2014). In addition, variants with a frequency above 1% or present in a homozygous state in the ExAC browser were excluded as potential pathogenic variants. Using standard settings in PLINK v1.07, the pairwise population concordance (PPC) and runs of homozygosity were determined (Purcell et al., 2007).

In **Family D**, sequencing was performed within the Radboudumc Innovative diagnostics programme. WGS was performed on 5 µg genomic DNA from the affected individual (D-V:3), using Agilent Sure Select Human whole exome kit V.6 (Agilent Technologies, Santa Clara, California, USA) for enrichment. Sequencing was undertaken on a PE 150 HiSeq machine (Illumina, San Diego, California, USA). Read alignment and variant calling were performed with GATK using default parameters with the human genome assembly hg19 (GRCh37) as reference. A stepwise filtering procedure was implemented starting with filtering out any variants with a minor allele frequency ≥ 1% in control databases (ExAC, gnomAD, dbSNP, the 1000 Genomes Project human polymorphism database and EVS). In the second step only protein coding non-synonymous, stop gain, frameshift or splice variants

as well as intronic splice site variants within 20 bp vicinity of exon/intron boundaries were retained. Given the consanguinity of the family, homozygous variants were prioritized.

In **Family E**, the study was approved by Istanbul University Cerrahpasa Medical Faculty Ethics Committee (83045809). WES was performed on 1 μg genomic DNA from the proband (E-III:2). Genomic DNA was captured using IDT xGen Exome Panel (Integrated DNA Technologies, Coralville, IA, USA), followed by 101 base paired-end sequencing on the Illumina NovaSeq 6000 at the Yale Center of Mendelian Genome. Primary analysis- sample de-multiplexing- was performed using Illumina's CASAVA 1.8.2 software suite. Sequences were aligned to the human genome GRCh37/hg19 with BWA-MEM (McKenna et al., 2010). Further processing was performed according to the best practices of the Genome Analysis Toolkit. Variant calls were made with GATK Haplotype Caller and annotated with ANNOVAR (Wang et al., 2012) plus in-house programs.

Variants were filtered for homozygous and compound heterozygous variants which had high quality sequence reads (pass GATK Variant Score Quality Recalibration (VSQR), a genotype quality (GQ) ≥ 20 and MAF $\leq 10^{-3}$ across all samples in ExAC, 1000 genomes and our in-house database which includes 635 samples from Turkish population). Loss-of-function variants (stop gains, stop losses, frameshift insertions and deletions, and canonical splice site mutations) were classified as damaging. Other coding variants were prioritized according to the amino acid conservation across species, and pathogenicity interpretation of prediction programs (CADD, Polyphen and SIFT).

In **Family F**, target enrichment was performed on genomic DNA from the proband (F-IV:1) using the SureSelect Human All Exon 50Mb Kit V7 (Agilent Technologies), followed by sequencing on the HiSeq4000 platform (Illumina). 97 % of the exome kit was covered at least 10x and the average sampling depth was 66x. Reads were aligned to the reference human genome (hg19/b37, UCSC assembly) using BWA (Li and Durbin, 2009) v. 0.7.13-r1126.

Single nucleotide variants and small insertions and deletions were detected with the HaplotypeCaller algorithm in the GATK module (McKenna et al., 2010). The detected variants were annotated and filtered by Mendelics in-house annotation software. A filtering strategy was performed using GnomAD 2.0.2 frequency ≤ 0.5% for homozygous and bialellic variants. The same threshold was used for 1K Genomes database. For heterozygous variants, the threshold was 0% for both databases. Genotyping variants with score less than 100 were ignored. Synonymous and intronic variants that were not included in ClinVar or in splicing site were ignored. Additional visual inspection was performed to reduce false positives.

mRNA analysis

mRNA analysis was performed to study the consequences of the splice variant identified in Family A. RNA was extracted from lymphoblastoid cell lines using the RNeasy PLUS mini kit (Qiagen, Courtaboeuf, France). Reverse transcription was performed using the Expand RT kit (Roche Applied Science, Meylan, France). Due to low expression, a nested PCR approach was used using the Taq CORE 10 NH4(SO4)2 kit (MP Biomedicals, Illkirch, France).

External primers: GTGGGCTGCTCATGTCCAGG and CAGATCCTGGCCCAGTCTTTC

Internal primers: GAACCCTCACAAGATGATGG et CTCCATCATCAGGGCTTTGA

The TOPO TA Cloning pCR™2.1-TOPO® vector kit (Life Technologies) was used to separate the three different PCR products. Sanger sequencing was performed after PCR amplification of 8 clones using the ABI Prism BigDye Terminator V3.1 kit (Life Technologies, Carlsbad, CA, USA) on a 3500DX instrument. (Thermo Fisher Scientific, WALTHAM, MA, USA). All steps were performed according to manufacturers' instructions.

2. Supplementary Results

Family A

In Family A, array-CGH was normal. WES was performed on DNA from peripheral blood from A-III:1 Non-synonymous, splice sites and indel variants were kept for analysis. Given the consanguinity in this family (Figure 1A), the analysis focused on homozygous variants. Among the 6 remaining variants, the c.1414-1G>C variant in *GAD1* was the only one predicted to truncate the protein (Supplementary Figure 1A) (Table below). The *GAD1* variant was absent from gnomAD control databases and predicted to be deleterious by several *in silico* prediction scores (DANN, GERP, MutationTaster). In addition, *in silico* splicing programs (SSF-like, MaxEntScan, and GeneSplicer) predicted the variant to abolish the acceptor splice-site. mRNA nested PCR amplification was performed after reverse transcription and resulted in three distinct PCR products. TOPO-cloning was used to characterize all different products. Exon 15 was completely absent from the smallest product (c.1414_1522del) while the largest one had a conserved exon 15 and 76 bases of intron 14 that were retained c.1413_1414ins ACAGGGACAGCATAGCCTTCCCAAATGCTCATCACAGGGAAATGCAACCACAAA CATGACTTTTCTCTTTAAAACAG. A third minor product was shown to be deleted of first 7 bases of exon 16. The normal isoform was absent and all three products were leading to a premature truncation of the protein, p.(Gly472Trpfs*72). Sanger sequencing confirmed the homozygous variant in both affected siblings (A-III:1 and A-III:2), and their unaffected parents were heterozygous carriers. It was classified as pathogenic according to the ACMG guidelines (Richards et al., 2015). Contribution of the SZT2 variant to the phenotype could not be formally ruled out, but based on phenotype comparisons and bioinformatic predictions we considered it as a variant of unknown significance.

Homozygous candidate variants for Family A			
**Gene**	Transcript	cDNA variant	Protein variant
MACF1	NM_012090.5	c.4894C>T	p.Leu1632Phe
MACF1	NM_012090.5	c.4949C>T	p.Thr1650Ile
SZT2	NM_015284.3	c.3390G>T	p.Lys1130Asn
KDM4A	NM_014663.2	c.1040C>T	p.Thr347Met
MYO3B	NM_138995.4	c.1869_1874delACATCA	p.His624_Gln625del
GAD1	NM_000817.2	c.1414-1G>C	

Family B

Data from WES on DNA from the proband (B-IV:4) was analyzed through the use of the Ingenuity Variant Analysis (IVA) software (Qiagen, Hilden Germany). The filtering strategy concentrating on homozygous coding variants in known congenital and neurogenetic disease genes revealed only a novel homozygous three base-pair deletion in exon 7 of *GAD1,* c.695_697delAGA (Supplementary Figure 1B), leading to the deletion of a lysine at position 232 P.(Lys232del), highly conserved residue across species (Supplementary Figure 2A). The variant was not present in any control databases (1000 genomes, ExAC/GnomAD browsers). *In silico* analysis predicted the p.Lys232del variant to be disease causing (MutationTaster). In addition, *in silico* prediction with the SpliceAid2 indicated that the three-base-pair AGA at position c.695_697 of *GAD1* mRNA is part of consensus binding sequences for seven different splicing factors. Consequently, the deletion of the AGA is predicted to result in a splice site loss, leading to a mRNA frameshift and subsequently to a premature termination codon. Sanger sequencing in all available family members confirmed segregation of the variant with the disease phenotype (Supplementary Figure 1B). The unaffected parents (B-III:1 and B-III:2) and one unaffected sibling (B-IV:2) were heterozygous carriers of the *GAD1* variant (Supplementary Figure 1B).

Family C

Whole genomic sequencing (WGS) was performed on DNA extracted from both healthy parents (C-II:1 and C-II:2) and the affected siblings (C-III:1 and C-III:2) of Family C (Supplementary Figure 1C). Data was generated, annotated, and variants prioritized as described before (Hardies et al., 2015). In addition, variants with a frequency above 1% or present in a homozygous state in the ExAC browser were excluded as potential pathogenic variants. Using standard settings in PLINK v1.07, the common ancestry of the parents was confirmed by a pairwise population concordance (PPC) value of 1, and the identification of 32 different homozygous regions covering almost 100 Mb genome wide. Homozygosity mapping combined with our filtering strategy resulted in the prioritization of six candidate variants (Table below). The two siblings (C-III:1 and C-III:2) were homozygous for a frameshift mutation in *GAD1*, c.812_816delTTAAG **(**Supplementary Figure 1C). The five-base pair deletion is predicted to result in a premature stop codon, p.(Val271Aspfs*9). This variant was not present in control databases (ExAC, GnomAD). It was the only loss-of-function mutation, predicted damaging by *in silico* tools, and located in the only gene with high expression levels in brain. Sanger sequencing confirmed segregation of the variant with the disease phenotype. The unaffected parents (C-II:1 and C-II:2) were heterozygous carriers of the c.812_816delTTAAG *GAD1* variant (Supplementary Figure 1C).

**Homozygous candidate variants for Family C**			
**Gene**	Transcript	cDNA variant	Protein variant
CLCNKB	NM_001165945	c.G1124A	p.R375H
PKP1	NM_001005337	c.C1787T	p.S596F
PAPOLB	NM_020144	c.A1126G	p.I376V
LRRC37B	NM_052888	c.A1863T	p.Q621H
COG1	NM_018714	c.T1036C	p.Y346H
GAD1	NM_000817.2	c.812_816delTTAAG	p.Val271Aspfs*9.

Family D

Data from WES on DNA from the proband (D-V:3) of Family D revealed a homozygous substitution in *GAD1* (c.1591C>T) leading to premature stop codon, p.(Arg531*) (Figure 3D). Sanger sequencing confirmed segregation of the variant with the disease. Both affected siblings (D-V:2 and D-V:3) were homozygous. The parents (D-IV:1 and D-IV:2) and one unaffected sibling (D-V:1) were heterozygous carriers of the *GAD1* variant (Supplementary Figure 1D). The *GAD1* c.1591C>T variant is present at very low frequency in the GnomAD (MAF: 3.98e-6). The variant was not identified in the homozygous state in any database.

**Homozygous candidate variants for Family D**			
**Gene**	Transcript	cDNA variant	Protein Variant
RNF19B	NM_001127361	c.A185C	p. Q62P
GAD1	NM_000817	c.C1591T	R531X
RAPGEF4	NM_001282901	c.A1055G	N352S
TTN	NM_003319	c.G58226A	p.R19409H
TTN	NM_133379	c.A10388G	p.H3463R
DNAH7	NM_018897	c.T10697C	p. F3566S
CFAP44	NM_001164496	c.G1426A	p.V476M
HLA-DRB5	NM_002125	c.G373T	p.E125X
HLA-DQA1	NM_002122	c.A295G	p.M99V
AGAP3	NM_001042535	c.A83G	p.Q28R
AGAP3	NM_001042535	c.T89G	p.L30R
WDR87	NM_031951	c.T6113A	p F2038Y
LOC400863	NM_001288961	c.C377T	p.S126F

Family E

WES was performed on DNA from the proband (E-III:2). The mean depth of the coverage was 69.3 with greater than 15x coverage in 98.6% of the target region. WES revealed a total of 32515 variants. After filtering, 14 homozygous, rare coding variants were retained (Table below). A homozygous substitution in *GAD1* (c.1591C>T) in exon 17 leading to premature stop codon p.(Arg531*) was identified (Supplementary Figure 1E). The *GAD1* variant was validated by Sanger sequencing and both parents were proven to be heterozygous

**Homozygous candidate variants for Family E**			
**Gene**	Transcript	cDNA variant	Protein Variant
JMJD4	NM_001161465	c.361G >A	p.A121T
SPRTN	NM_032018	c.1058G>A	p.S353N
CSRNP3	NM_024969	c.940G>A	p.A314T
GAD1	NM_000817	c.1591C>T	p.R531X
POLQ	NM_199420	c.4244C>T	p.P1415L
KIAA1549	NM_001164665	c.1031C>G	p.T344S
SPTBN5	NM_016642	c.7921G>A	p.G2641S
PARG	ENST00000425119.2	c..1076C>T	p.S359F
DUOX2	NM_014080	c.346G>A	p.V116M
GDE1	NM_001324067	c.637T>G	p.F213V
TANC2	NM_025185	c.1370A>G	p.Y457C
MZF1	NM_003422	c.970C>T	p.R324W
NLGN4X	NM_001282146	c.71A>G	p.N24S
CNKSR2	ENST00000279451.4	c.1394-7T>G	

Family F

WES was performed on DNA from the proband (F-IV:1) of Family F. After the filtering strategy, a total of eleven homozygous missense, splice site or indel rare variants were identified (Table below). A homozygous missense variant in *GAD1* (c.1525G>A) (Supplementary Figure 1F), changing the conserved glutamate at position 509 to lysine p.(Glu509Lys), was identified (Supplementary Figure 2B). The *GAD1* variant was validated by Sanger sequencing and both parents were heterozygous carriers for the variant.

**Homozygous candidate variants for Family F**			
**Gene**	Transcript	cDNA variant	Protein variant
MROH8	ENST00000441008	c.50_51insAGTGCCGGCCGCGGGGCCCTGTCTATAAG	p.Ser17_His18fs
KCNN3	ENST00000271915	c.241_242insAGCAGCAGCAGCAGC	p.Pro81_Pro82insGlnGlnGlnGlnGlnPro
GAD1	ENST00000358196	c.1525G>A	p.Glu509Lys
HOXD13	ENST00000392539	c.202G>C	p.Ala68Pro
TTN	ENST00000460472	c.43867T>G	p.Ser14623Ala
ATXN1	ENST00000436367	c.626_627insGCAGCA	p.His209_Gln210insGlnGlnHis
KRT4	ENST00000458244	c.199_200insCCGGCGGCTTCGGAGCTGGTTTCGGCACTGGTGGCTTTGGTG	p.Gly67_Gly68insAlaGlyGlyPheGlyAlaGlyPheGlyThrGlyGlyPheGlyGly
EPG5	ENST00000282041	c.4891G>A	p.Ala1631Thr
LOXHD1	ENST00000579038	c.52A>G	p.Ser18Gly
SMAD7	ENST00000591805	c.19A>G	p.Thr7Ala
SLC27A1	ENST00000442725	c.1835G>A	p.Arg612His

3. Supplementary References

DePristo, M.A., et al., 2011. A framework for variation discovery and genotyping using next-generation DNA sequencing data. Nat Genet. 43**,** 491-8.

Hardies, K., et al., 2015. Recessive loss-of-function mutations in AP4S1 cause mild fever-sensitive seizures, developmental delay and spastic paraplegia through loss of AP-4 complex assembly. Hum Mol Genet. 24**,** 2218-27.

Kariminejad, A., et al., 2016. Lethal multiple pterygium syndrome, the extreme end of the RYR1 spectrum. BMC Musculoskelet Disord. 17**,** 109.

Li, H., Durbin, R., 2009. Fast and accurate short read alignment with Burrows-Wheeler transform. Bioinformatics. 25**,** 1754-60.

Li, H., et al., 2009. The Sequence Alignment/Map format and SAMtools. Bioinformatics. 25**,** 2078-9.

McKenna, A., et al., 2010. The Genome Analysis Toolkit: a MapReduce framework for analyzing next-generation DNA sequencing data. Genome Res. 20**,** 1297-303.

Purcell, S., et al., 2007. PLINK: a tool set for whole-genome association and population-based linkage analyses. Am J Hum Genet. 81**,** 559-75.

Richards, S., et al., 2015. Standards and guidelines for the interpretation of sequence variants: a joint consensus recommendation of the American College of Medical Genetics and Genomics and the Association for Molecular Pathology. Genet Med. 17**,** 405-24.

Schubert, J., et al., 2014. Mutations in STX1B, encoding a presynaptic protein, cause fever-associated epilepsy syndromes. Nat Genet. 46**,** 1327-32.

Sherry, S.T., et al., 2001. dbSNP: the NCBI database of genetic variation. Nucleic Acids Res. 29**,** 308-11.

Wang, K., Li, M., Hakonarson, H., 2010. ANNOVAR: functional annotation of genetic variants from high-throughput sequencing data. Nucleic Acids Res. 38**,** e164.

Wang, Y., et al., 2012. Expression of the inclusion body myopathy 3 mutation in Drosophila depresses myosin function and stability and recapitulates muscle inclusions and weakness. Mol Biol Cell. 23**,** 2057-65.

4. Supplementary Figure legends

Supplementary Figure 1. Genetic findings

Results from WES/WGS as presented using Integrated Genome Viewer (left panels) and Sanger sequencing (right panels). *GAD1* variants were homozygously present in all probands and available affected family members. Parents and available unaffected siblings were heterozygous for variants **(A)** c.1414-1G>C variant in Family A. **(B)** Three-base deletion c.695_697delAGA in Family B. **(C)** Homozygous five-base pair deletion c.812_816delTTAAG in two affected siblings (C-III:1 and C-III:2) in Family C. Both healthy parents (C-II:1 and C-II:2) are carriers of the frameshift mutation. (**D**) Substitution c.1591C>T in Family D. (**E**) c.1591C>T variant in Family E. (**F**) Presence of c.1525G>A in Family F.

Supplementary Figure 2. Multiple sequence alignment

Multiple sequence alignment confirms that the (**A**) p.(Lys232del) and (**B**) p.(Glu509Lys) *GAD1* substitutions affect evolutionarily conserved residues (shaded).

Supplementary Figure 3. Immunoblot analysis of *in vitro* protein expression of WT- and p.Lys232del-GAD67

(**A**) Western blot analysis demonstrates that the mutated p.Lys232del-GAD67 was expressed at a comparable levels as WT-GAD67. (**B**) Gel electrophoresis indicates comparable levels of p.Lys232del-GAD67 and WT-GAD67.

Supplementary Figure 4. Structural modeling of wildtype GAD67 and the p.(Glu509Lys) variant.

(**A**) GAD67 structure model generated using HOPE bioinformatics tools. Overview of the protein in ribbon-presentation. The protein is colored by element; α-helix=blue, β-strand=red, turn=green, 3/10 helix=yellow and random coil=cyan. Other molecules in the complex are colored grey when present. (**B**) The mutated side chain is shown in magenta. (**C-E**) Close-up of the mutated position within the protein structure model taken from different angles (green: wildtype, red: mutated).

Supplementary Figure 5. Schematic visualization of *GAD1* structure and the variants. Localization of identified variants at the *GAD1* genomic level (upper part) and the GAD67 protein level (lower part).
