# Supplementary material for: Bi-allelic GAD1 variants cause a neonatal onset syndromic developmental and epileptic encephalopathy
Source: Brain. 2020 Apr 13;143(5):1447–61. doi: 10.1093/brain/awaa085 (PMC7241960; doi:10.1093/brain/awaa085)
Supplement: awaa085_Supplementary_Data [file awaa085_supplementary_data.zip › awaa085-suppl_data/brain-2019-01328-File009.pdf]

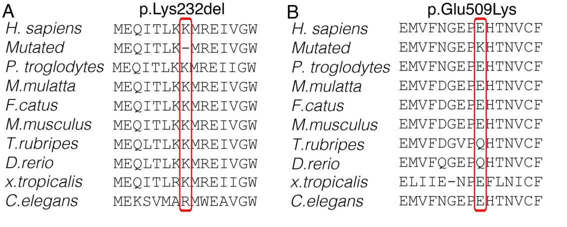
A
p.Lys232del
H. sapiens MEQITLKKMREIVGW
Mutated MEQITLK-MREIVGW
P. troglodytes MEQITLKKMREIIGW
M.mulatta MEQITLKKMREIVGW
F.catus MEQITLKKMREIVGW
M.musculus MEQITLKKMREIVGW
T.rubripes MEQLTLKKMREIVGW
D.rerio MEQLTLKKMREIVGW
x.tropicalis MEQITLRKMREIIGW
C.elegans MEKSVMARMWEAVGW
B
p.Glu509Lys
H. sapiens EMVFNGEPEHTNVCF
Mutated EMVFNGEPKHTNVCF
P. troglodytes EMVFNGEPEHTNVCF
M.mulatta EMVFDGEPEHTNVCF
F.catus EMVFDGEPEHTNVCF
M.musculus EMVFDGEPEHTNVCF
T.rubripes EMVFDGVPQHTNVCF
D.rerio EMVFQGEPQHTNVCF
x.tropicalis ELIIE-NPEFLNICF
C.elegans EMVFNGEPEHTNVCF
