# Supplementary figures and images for: Bi-allelic GAD1 variants cause a neonatal onset syndromic developmental and epileptic encephalopathy
Source: Brain. 2020 Apr 13;143(5):1447–61. doi: 10.1093/brain/awaa085 (PMC7241960; doi:10.1093/brain/awaa085)

**A** p.Lys232del-GAD67 WT-GAD67

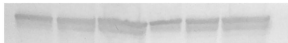

**B** p.Lys232del-GAD67 WT-GAD67

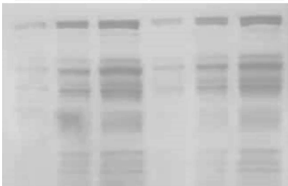

Supplement: awaa085_Supplementary_Data [file awaa085_supplementary_data.zip › awaa085-suppl_data/brain-2019-01328-File010.pdf]

A

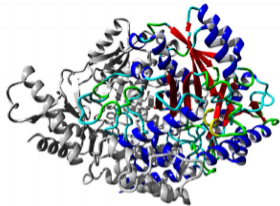

B

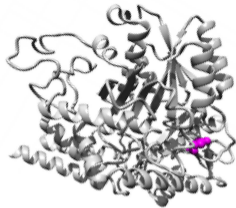

C

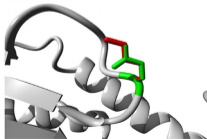

D

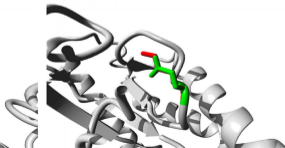

E

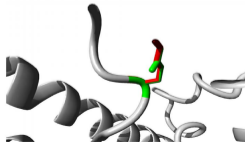

Supplement: awaa085_Supplementary_Data [file awaa085_supplementary_data.zip › awaa085-suppl_data/brain-2019-01328-File011.pdf]

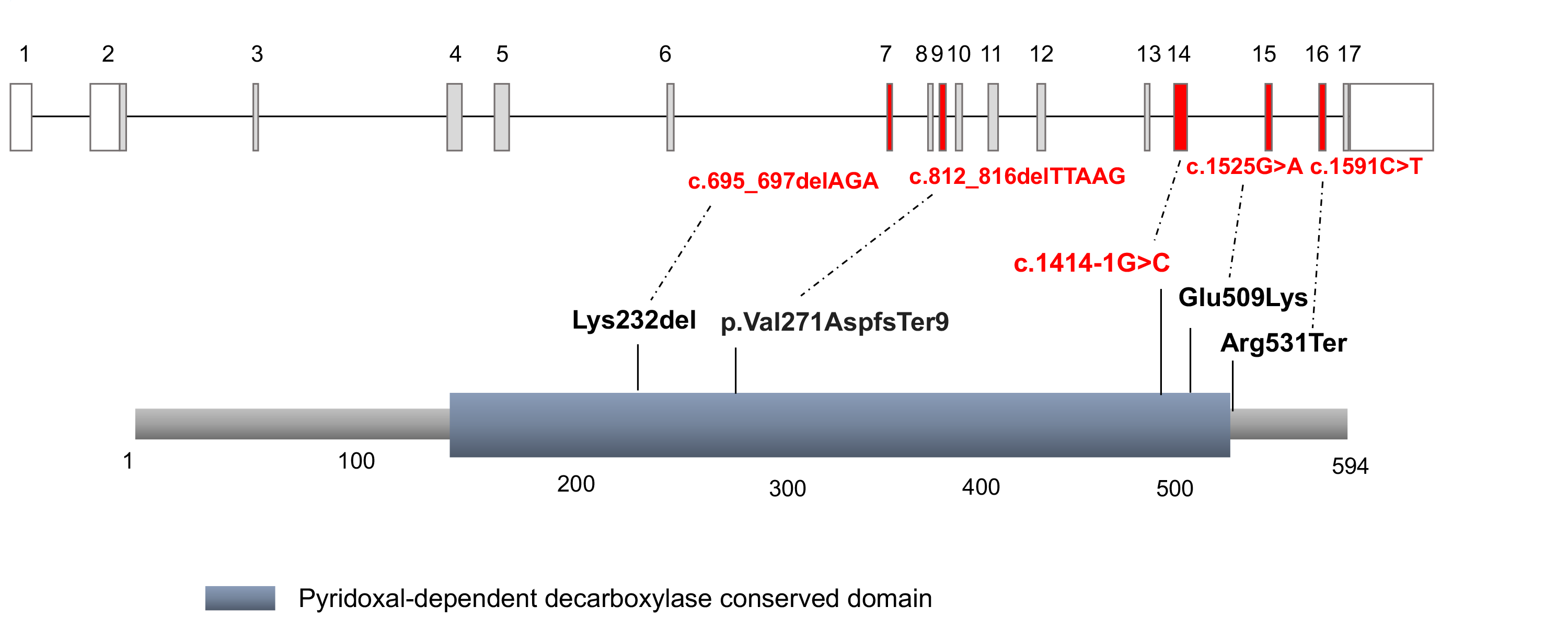

Supplement: awaa085_Supplementary_Data [file awaa085_supplementary_data.zip › awaa085-suppl_data/brain-2019-01328-File012.tif]

**A**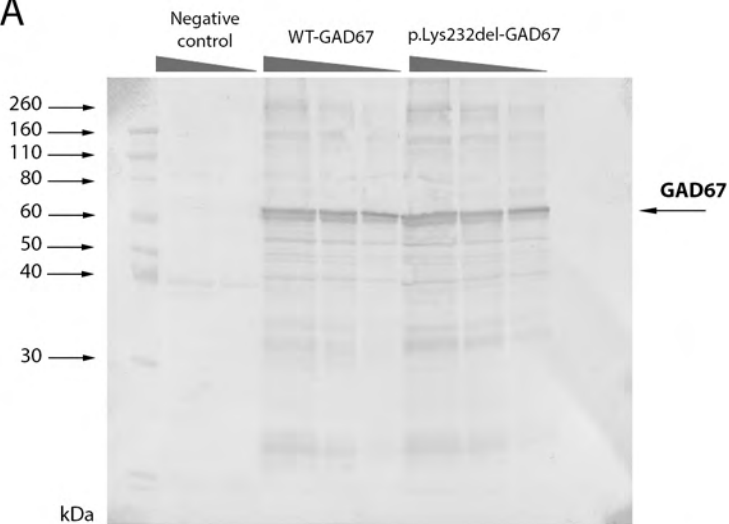**B**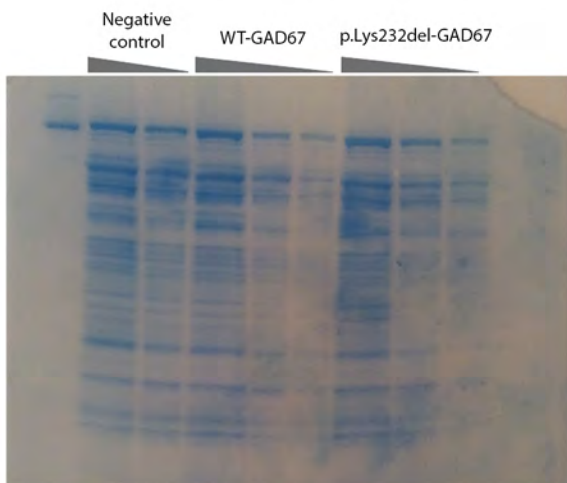

Supplement: awaa085_Supplementary_Data [file awaa085_supplementary_data.zip › awaa085-suppl_data/brain-2019-01328-File013.pdf]
