# Supplementary material for: Bi-allelic GAD1 variants cause a neonatal onset syndromic developmental and epileptic encephalopathy
Source: Brain. 2020 Apr 13;143(5):1447–61. doi: 10.1093/brain/awaa085 (PMC7241960; doi:10.1093/brain/awaa085)
Supplement: awaa085_Supplementary_Data [file brain-2019-01328-File008.pdf › awaa085-suppl_data/brain-2019-01328-File008.pdf]

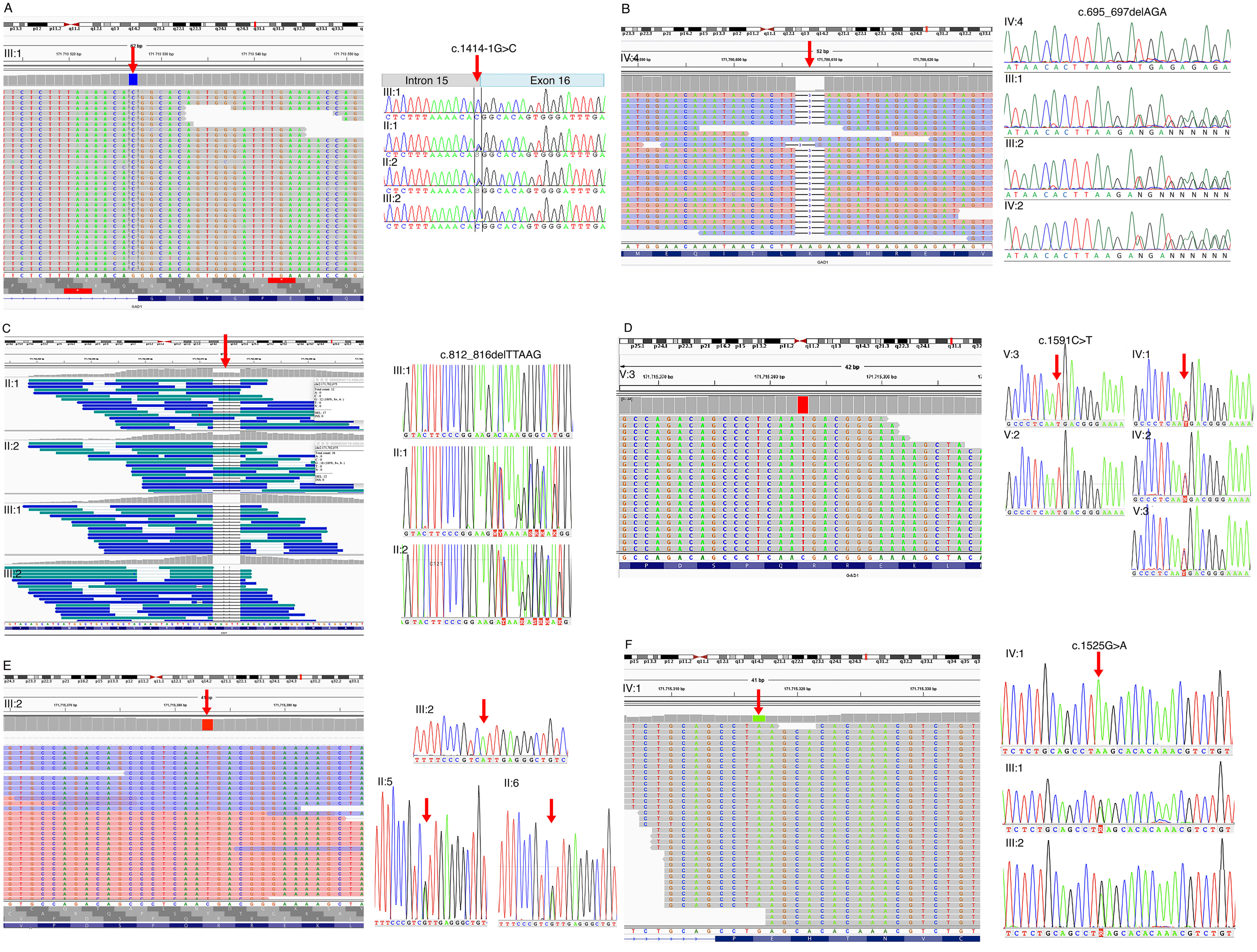
A
III:1
c.1414-1G>C
Intron 15
Exon 16
III:1
II:1
II:2
III:2
GAD1
B
c.695_697delAGA
IV:4
52 bp
III:1
III:2
IV:2
GAD1
C
c.812_816delTTAAG
II:1
II:2
III:1
III:2
D
c.1591C>T
V:3
42 bp
IV:1
V:2
IV:2
V:3
GAD1
E
III:2
II:5
II:6
F
c.1525G>A
IV:1
41 bp
III:1
III:2
